# Pharmacogenomics deliberations of 2-deoxy-d-glucose in the treatment of COVID-19 disease: an in silico approach

**DOI:** 10.1007/s13205-022-03363-4

**Published:** 2022-09-21

**Authors:** Navya B. Prabhu, Chigateri M. Vinay, Kapaettu Satyamoorthy, Padmalatha S. Rai

**Affiliations:** 1 Department of Biotechnology, Manipal School of Life Sciences, Manipal Academy of Higher Education, Manipal, India; 2 Department of Cell and Molecular Biology, Manipal School of Life Sciences, Manipal Academy of Higher Education, Manipal, India

**Keywords:** 2-Deoxy-d-glucose (2-DG), SARS-CoV-2, COVID-19, Single nucleotide polymorphisms, Drug response

## Abstract

The outbreak of COVID-19 caused by the coronavirus (SARS-CoV-2) prompted number of computational and laboratory efforts to discover molecules against the virus entry or replication. Simultaneously, due to the availability of clinical information, drug-repurposing efforts led to the discovery of 2-deoxy-d-glucose (2-DG) for treating COVID-19 infection. 2-DG critically accumulates in the infected cells to prevent energy production and viral replication. As there is no clarity on the impact of genetic variations on the efficacy and adverse effects of 2-DG in treating COVID-19 using in silico approaches, we attempted to extract the genes associated with the 2-DG pathway using the Comparative Toxicogenomics Database. The interaction between selected genes was assessed using ClueGO, to identify the susceptible gene loci for SARS-CoV infections. Further, SNPs that were residing in the distinct genomic regions were retrieved from the Ensembl genome browser and characterized. A total of 80 SNPs were retrieved using diverse bioinformatics resources after assessing their (a) detrimental influence on the protein stability using Swiss-model, (b) miRNA regulation employing miRNASNP3, PolymiRTS, MirSNP databases, (c) binding of transcription factors by SNP2TFBS, SNPInspector, and (d) enhancers regulation using EnhancerDB and HaploReg reported A2M rs201769751, PARP1 rs193238922 destabilizes protein, six polymorphisms of XIAP effecting microRNA binding sites, *EGFR* rs712829 generates 15 TFBS, *BECN1* rs60221525, *CASP9* rs4645980, *SLC2A2* rs5393 impairs 14 TFBS, *STK11* rs3795063 altered 19 regulatory motifs. These data may provide the relationship between genetic variations and drug effects of 2-DG which may further assist in assigning the right individuals to benefit from the treatment.

**Supplementary Information:**

The online version contains supplementary material available at 10.1007/s13205-022-03363-4.

## Introduction

The outbreak of coronavirus disease 2019 (COVID-19) caused by the new coronavirus (SARS-CoV-2) infection has prompted worldwide attempts to develop efficient molecules to treat the disease and symptoms (Samantaray et al. 2021). However, developing novel molecules culminating in translation against infections can be laborious, time-consuming, and expensive (Paul et al. 2010). Thus, identifying the therapeutically effective entity against the disease from a pre-existent clinically approved repository of molecules may be advantageous (Ciliberto et al. 2020).

Virus infections such as SARS-CoV-2 reprogram the host cells to consume more glucose and upregulate metabolic activities such as glycolysis, akin to the Warburg effect and alter glycosylation to survive, replicate, and transmit infections (Mullen et al. 2021). Similar to glucose, internalization of 2-DG is facilitated by glucose transporters followed by its phosphorylation into inactive metabolite, 2-deoxyglucose-6-phosphate. This glucose deprivation in the cells leads to reduced proliferation and induction of apoptosis (Schmidt and O’Donnell 2021).

2-Deoxy-d-glucose (2-DG) has been tested to inhibit glycolysis and hence SARS-CoV-2 replication in monocytes and epithelial cells consequently leading to increased HIF-1α and reduced inflammatory mediators (Pliszka and Szablewski 2021; Medini et al. 2021; Codo et al. 2020). It was reported that 2-DG prevents viral replication by hindering virus DNA polymerase (Codo et al. 2020; Liu et al. 2021) attaching to specified receptors on the cell surface and obstructing viral invasion into the target cells; blocking viral protein synthesis, obstructing delayed phases of virus assembly (Codo et al. 2020). The metabolic processes such as glycolysis in the cytoplasm and glycosylation in the endoplasmic reticulum can be interrupted using glucose mimics such as 2-deoxy-D-glucose (2-DG) (Xi et al. 2014). By impeding viral replication, high energy requirements, and viral assembly, it could be a potential therapeutic candidate (Khurana et al. 2022).

The reports from the different phases of clinical trials have shown that 2-DG aids in improving the health status of severely Covid-19-infected individuals and decreases oxygen therapy dependency. It was found that a large number of 2-DG-treated patients reported negative within 5 days (Goel 2021; Wang et al. 2021). 2-DG as an anti-viral agent has previously been reported wherein the inhibition of replication of enveloped viruses such as herpes simplex virus (Courtney et al. 1973), measles virus, respiratory syncytial virus (Hodes et al. 1975), Semiliki forest virus, and Sindbis virus (Kaluza et al. 1972) are demonstrated. In an in vivo study, 2-DG inhibited rhinovirus load and inflammation in mice (Gualdoni et al. 2018). Several proteins such as non-structural protein 1 (Nsp1), RNA-dependent RNA polymerase, 3CLpro are the attractive targets involved in COVID-19 treatment (Singh et al. 2021, 2022). In silico analysis suggested efficient binding of 2-DG with SARS-CoV-2 viral main protease 3CLpro and NSP15 endoribonuclease (Balkrishna et al. 2020). As considerable knowledge on molecular interaction between 2-DG and SARS-CoV-2 and drug response is lacking, there is an absolute requisite to integrate the information from 2DG interacting genes by in silico analysis. The genes and their products are regulated by various mechanisms that involve correlation between many processes, metabolic pathways, and regulatory factors (Vohra et al. 2021). One prevalent form of gene variants is single nucleotide polymorphisms (SNPs), where two different bases appear at a remarkable rate in human diversity (Prabhu et al. 2021). The genetic profiling based on the identified and functionally characterized SNPs is considered a “fingerprint”, possibly used to determine the risk of disease susceptibility and drug response (Shastry 2007). Many variants residing in non-coding and non-regulatory sequences are functionally silent. However, few SNPs alter the structure and function of the protein. The role of functional SNPs, which can alter the regulation and structure of the protein in relation to the effects of 2-DG, is not well understood. These functional SNPs are considered an ideal substrate for the human population in health and illness (Alwi 2005).

Hence, the current study is aimed to investigate the influence of functional or regulatory SNPs on the potency and pernicious effect of 2-DG. Therefore, the main purpose of the research was to examine the impact of SNPs in the 2-DG interacting pathway genes by interrogating various bioinformatics resources and assessed the influence of SNPs on the protein stability, miRNA regulation, and *cis*-acting elements to evolve a relationship for pharmacogenomics purposes.

## Materials and methods

### Identification of interacting genes of 2-DG

The interacting genes of 2-DG were retrieved from the Comparative Toxicogenomic Database (CTD) (Grondin et al. 2021) using the parameter named chemical-gene interaction in *Homo sapiens.* UniProt database (Uniprot Consortium 2021) was used to retrieve the data of all the 2-DG interacting gene families, and further, these data were utilized for downstream analysis.

### Pathway interaction among 2-DG interacting genes

The 2-DG interacting genes were subjected to the Cytoscape tool v3.0 Software ClueGO v2.5.8 (Bindea et al. 2009) was employed to identify the networks in the degree sorted circular layout to interpret the biological function of the selected genes. The distinct ontologies such as molecular function, pathways, and human diseases were used in the framework, and the GO terms were connected using kappa statistics based on the overlapping genes.

### Retrieval and characterization of SNPs

For the selected genes, SNPs were retrieved by preferring the option variant table in the Ensembl genome browser (m.ensembl.org). The retrieved SNPs were further classified into missense variants, 5′-UTR variants, 3′-UTR variants, synonymous SNPs, intronic SNPs, splice donor, splice acceptor variants, splice region SNPs, stop retained SNPs, stop-loss SNPs, stop-gained SNPs, and non-coding transcript exon variants. Among these, missense SNPs were considered for further functional analysis.

### In silico prediction of missense variants functional impacts

The selected missense variants were scrutinized utilizing six diverse tools with mutation score accessible in the Ensembl genome browser, and these included CADD (Combined Annotation-Dependent Depletion), Mutation assessor, SIFT (Sorting Intolerant from Tolerant), Revel (Rare exome variant ensemble learner), MetaLR, and PolyPhen-2 (Polymorphism Phenotyping). The SNPs characterized as “deleterious” in all the tools were carefully chosen and evaluated for their effect on protein structure and stability.

### Protein modeling and mutation effect on protein stability

To interpret the effect of deleterious SNPs on protein structure, we predicted the native and mutant forms by protein modeling. The predicted model of the native form was available from the AlphaFold protein structure database (Jumper et al. 2021), and the mutant form of the protein structure was modeled using an automated protein structure homology-modeling server, SWISS-MODEL via Expasy webserver (Waterhouse et al. 2018), by considering the native predicted model as a template. The alteration in the hydrophilicity or hydrophobicity for the deleterious SNPs due to the amino acid change is presented using the hydropathy index (Kyte et al. 1982). The stability of the protein was determined based on point mutation using the CUPSAT mutation tool (Parthiban et al. 2006) of the 3D AlphaFold structure of variants retrieved from UniProt database. Using Swiss-PDB Viewer (Kaplan and Littlejohn 2001), the energy minimization using the steepest descent algorithm was performed for the mutated protein model with the corresponding amino acid substitution, compared with the native protein model, followed by total energy calculations. The root-mean-square deviation (RMSD) was calculated using align function from Pymol software to find the divergence in mutant form from the native form of the protein (Yuan et al. 2017).

### Prediction of functional microRNA target SNPs

The identified 2-DG-associated genes were deployed to predict the SNPs in the microRNA binding sites that were functional using three databases. These were microRNA-related Single Nucleotide Polymorphisms v3 (miRNASNP3) (Gong et al. 2015), PolymiRTS database (Bhattacharya et al. 2014), and miRNA-related SNPs (MirSNP) database (Liu et al. 2012). The MirSNP database was utilized to investigate the miRNA binding SNP locations and their consequences on the target position. Furthermore, the PolymiRTS database was employed to obtain the variants and their concomitant miRNAs at wild and mutant alleles and assessed their effect on the target gain/loss in the 3′-UTR using the miRNASNP3 database.

### SNPs at the transcription factor binding site (TFBS)

The shortlisted 2-DG interacting genes were utilized to obtain the SNPs in TFBS employing SNP2TFBS (Kumar et al. 2017). The parameter named annotated variants were employed to obtain the SNPs residing in the upstream and 5′-UTR regions. The SNPInspector in Genomatix Software Suite (https://www.genomatix.de/) was applied to predict if SNPs in TFBS generate or destroy the TF binding sites.

### Enhancers SNPs

The identified 2-DG-associated genes were further utilized to analyze the influence of SNPs residing in enhancers using EnhancerDB (Kang et al. 2019) and ENCODE laboratories software HaploReg version 4.1 (Ward and Kellis 2012). The search preference comprising gene was utilized in the EnhancerDB database to retrieve the enhancer SNPs of the shortlisted genes. Further, HaploReg v4.1 was used to evaluate the regulatory motifs of the enhancer SNPs that were altered.

## Results

### Identification of interacting genes for 2-DG

**Fig. 1:**
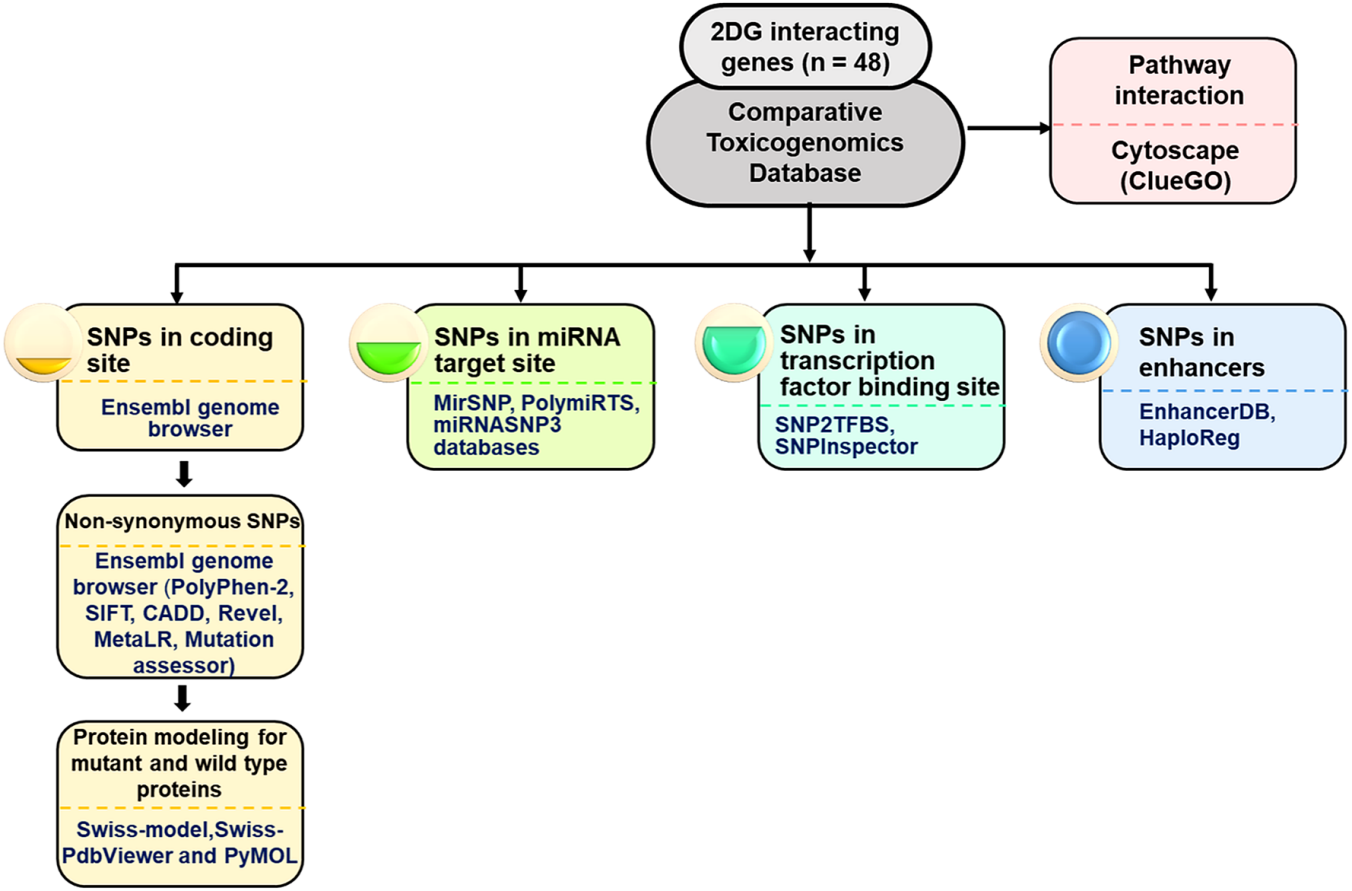
Schematic representation of in silico workflow of the study

We identified 48 interacting genes for 2-DG (Table 1) and plotted their position using the Circos ideogram. The depiction indicated the distribution of genes over 21 autosomes and X chromosome except for 13 autosome and Y chromosome (Fig. S1). The overview of plot shows 48 genes (from outer ring inwards), 5′-UTR SNPs, intronic SNPs, 3′-UTR SNPs, synonymous SNPs, missense variants, splice variants (splice region, splice donor, splice acceptor), start lost, stop-lost, stop-gained, stop-lost SNPs, inner most ring constitutes non-coding transcript exon variant and NMD transcript variant. The schematic illustration of in silico workplan is shown in Fig. 1.

**Table 1:** Details of selected 2DG interacting genes for downstream analysis

Sl. No.	Gene	Gene symbol	Family	Chromosome	Location	Strand
1	*Alpha-2-Macroglobulin*	*A2M*	Protease inhibitor	chr12	9,067,664–9,116,229	Minus
2	*Adiponectin, C1Q And Collagen Domain Containing*	*ADIPOQ*	Hormone	chr3	186,842,704–186,858,463	Plus
3	*Amyloid Beta Precursor Protein*	*APP*	Protease inhibitor	chr21	25,880,550–26,171,128	Minus
4	*Autophagy Related 7*	*ATG7*	–	chr3	11,272,309–11,564,652	Plus
5	*BCL2 Associated X, Apoptosis Regulator*	*BAX*	–	chr19	48,954,815–48,961,798	Plus
6	*Beclin 1*	*BECN1*	–	chr17	42,810,132–42,833,350	Minus
7	*BH3 Interacting Domain Death Agonist*	*BID*	–	chr22	17,734,138–17,774,770	Minus
8	*Caspase 3*	*CASP3*	Protease	chr4	184,627,696–184,649,509	Minus
9	*Caspase 9*	*CASP9*	Protease	chr1	15,490,832–15,526,534	Minus
10	*DNA Damage Inducible Transcript 3*	*DDIT3*	–	chr12	57,516,588–57,521,737	Minus
11	*Epidermal Growth Factor Receptor*	*EGFR*	Kinase	chr7	55,019,017–55,211,628	Plus
12	*Eukaryotic Translation Initiation Factor 4E Binding Protein 1*	*EIF4EBP1*	Protein synthesis inhibitor	chr8	38,030,534–38,060,365	Plus
13	*Erb-B2 Receptor Tyrosine Kinase 2*	*ERBB2*	Kinase	chr17	39,687,914–39,730,426	Plus
14	*FXYD Domain Containing Ion Transport Regulator 2*	*FXYD2*	Ion transport	chr11	117,800,844–117,828,698	Minus
15	*Glycogen Synthase Kinase 3 Beta*	*GSK3B*	Kinase	chr3	119,821,321–120,095,823	Minus
16	*H2A.X Variant Histone*	*H2AX*	–	chr11	119,093,854–119,095,467	Minus
17	*Hexokinase 2*	*HK2*	Kinase	chr2	74,834,126–74,893,359	Plus
18	*Heme Oxygenase 1*	*HMOX1*	Oxidoreductase	chr22	35,380,361–35,394,214	Plus
19	*Heat Shock Protein 90 Alpha Family Class A Member 1*	*HSP90AA1*	Protease	chr14	102,080,742–102,139,749	Minus
20	*Heat Shock Protein 90 Beta Family Member 1*	*HSP90B1*	Chaperone	chr12	103,930,107–103,953,931	Plus
21	*Heat Shock Protein Family A (Hsp70) Member 5*	*HSPA5*	Protease	chr9	125,234,853–125,241,382	Minus
22	*HtrA Serine Peptidase 2*	*HTRA2*	Protease	chr2	74,529,405–74,533,556	Plus
23	*Insulin Like Growth Factor 1 Receptor*	*IGF1R*	Kinase	chr15	98,648,539–98,964,530	Plus
24	*Interleukin 1 Beta*	*IL1B*	Cytokine receptors	chr2	112,829,751–112,836,843	Minus
25	*Insulin*	*INS*	Hormone	chr11	2,159,779–2,161,221	Minus
26	*Integrin Subunit Beta 1*	*ITGB1*	Integrin	chr10	32,887,273–33,005,792	Minus
27	*Potassium Voltage-Gated Channel Subfamily H Member 2*	*KCNH2*	Ion channel	chr7	150,944,956–150,978,321	Minus
28	*Microtubule Associated Protein 1 Light Chain 3 Alpha*	*MAP1LC3A*	–	chr20	34,546,823–34,560,345	Plus
29	*Microtubule Associated Protein 1 Light Chain 3 Beta*	*MAP1LC3B*	–	chr16	87,383,953–87,404,779	Plus
30	*Mitogen-Activated Protein Kinase 1*	*MAPK1*	Kinase	chr22	21,759,657–21,867,680	Minus
31	*Mitogen-Activated Protein Kinase 3*	*MAPK3*	Kinase	chr16	30,114,105–30,123,506	Minus
32	*Matrix Metallopeptidase 13*	*MMP13*	Protease	chr11	102,942,995–102,955,732	Minus
33	*Matrix Metallopeptidase 9*	*MMP9*	Protease	chr20	46,008,908–46,016,561	Plus
34	*Mechanistic Target Of Rapamycin Kinase*	*MTOR*	Kinase	chr1	11,106,535–11,273,497	Minus
35	*Nitric Oxide Synthase 2*	*NOS2*	Oxidoreductase	chr17	27,756,766–27,800,529	Minus
36	*Poly (ADP-Ribose) Polymerase 1*	*PARP1*	Glycosyltransferase	chr1	226,360,210–226,408,154	Minus
37	*Ribosomal Protein S6*	*RPS6*	Ribonucleoprotein	chr9	19,375,715–19,380,236	Minus
38	*Ribosomal Protein S6 Kinase B1*	*RPS6KB1*	Kinase	chr17	59,893,046–59,950,574	Plus
39	*Serpin Family B Member 5*	*SERPINB5*	–	chr18	63,476,958–63,505,085	Plus
40	*Solute Carrier Family 2 Member 1*	*SLC2A1*	Transport	chr1	42,925,353–42,958,893	Minus
41	*Solute Carrier Family 2 Member 2*	*SLC2A2*	Transport	chr3	170,996,341–171,026,743	Minus
42	*Solute Carrier Family 2 Member 3*	*SLC2A3*	Transport	chr12	7,919,230–7,936,187	Minus
43	*Superoxide Dismutase 2*	*SOD2*	Oxidoreductase	chr6	159,669,069–159,762,529	Minus
44	*Sequestosome 1*	*SQSTM1*	–	chr5	179,806,393–179,838,078	Plus
45	*Serine/Threonine Kinase 11*	*STK11*	Kinase	chr19	1,177,558–1,228,431	Plus
46	*Tumor Necrosis Factor*	*TNF*	Cytokine	chr6	31,575,565–31,578,336	Plus
47	*Tumor Protein P53*	*TP53*	Activator and repressor	chr17	7,661,779–7,687,538	Minus
48	*X-Linked Inhibitor of Apoptosis*	*XIAP*	Protease inhibitor	chr X	123,859,708–123,913,976	Plus

*SNPs* single nucleotide polymorphisms, *chr* chromosome

### Pathway interaction among 2-DG interacting genes

The interaction among 2-DG genes constituted a network after employing the statistical option Enrichment/Depletion test (two-sided hypergeometric test) (Fig. S2). The resulting network indicated 13 Kappa score groups such as apoptotic factor-mediated response, the intrinsic pathway for apoptosis, cytochrome C-mediated apoptotic response, interleukin-4, and interleukin-13 signaling, integration of energy metabolism, macroautophagy, purinergic signaling in leishmaniasis infection, ATF6 alpha activates chaperone genes, mTOR signaling, FOXO-mediated transcription, protease binding, collagen-binding and SARS-CoV infections (Fig. S2). It was found that 45.87% of the associated genes (*CASP3, CASP9, MAPK1, MAPK3, XIAP*) contributed to cytochrome C-mediated apoptotic response and 3.67% of the associated genes (*BECN1, FXYD2, GSK3B, HSP90AA1, ITGB1, MAP1LC3B*) contributed to SARS-CoV infections (Fig. S2).

### SNPs characterization

**Fig. 2:**
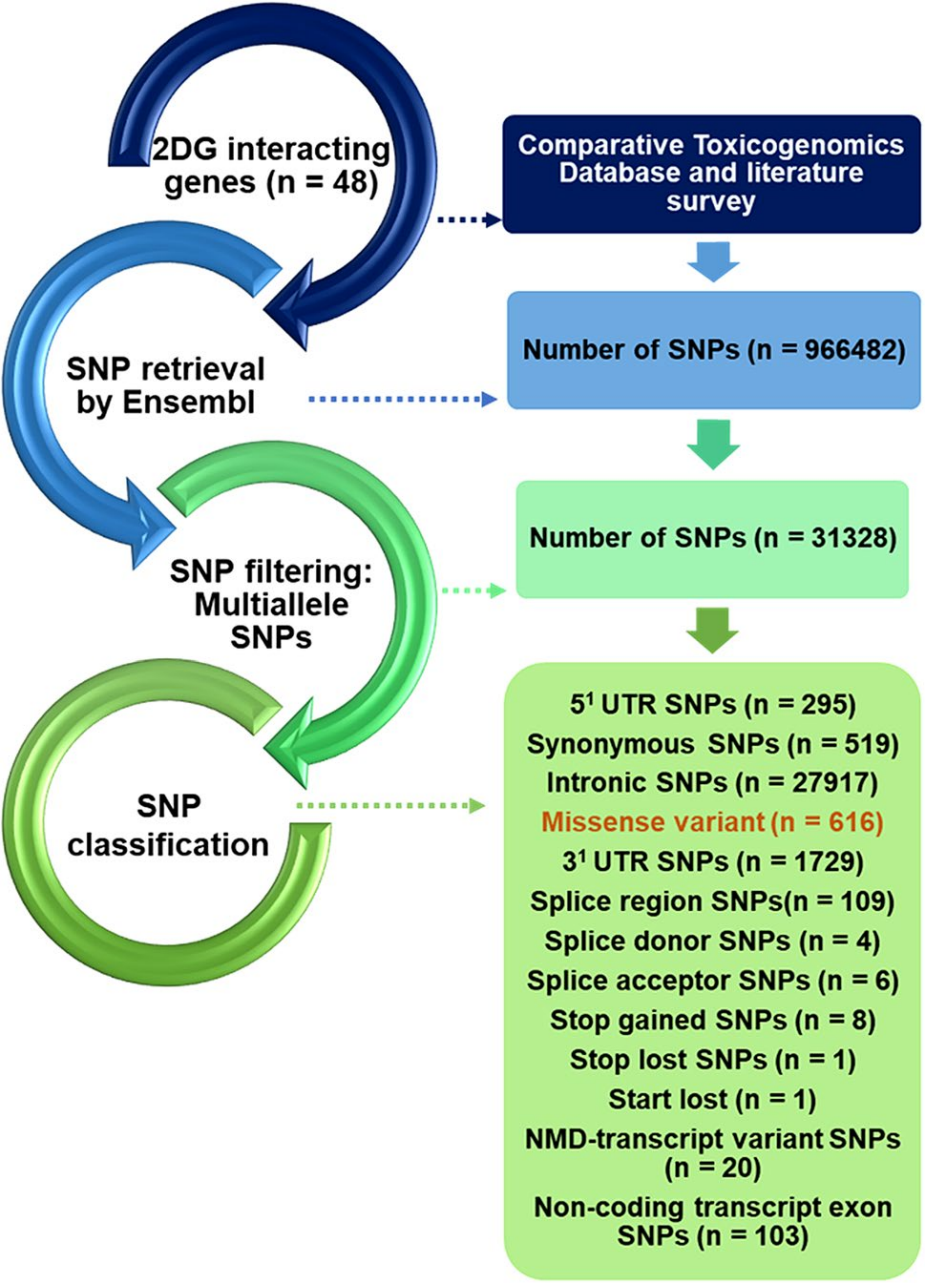
Schematic representation of in silico SNP search and characterization

A sum of 9,66,482 SNPs was obtained by using the Ensembl genome browser (m.ensembl.org) from human genome assembly GRCh38.p13 (1000 Genomes Project). The retrieved variants were mined which generated 1,04,034 SNPs. These shortlisted variants were further classified depending on their function. These SNPs were from 5′-UTR (295), intronic regions (27,917), 3′-UTR (1729), synonymous SNPs (519), splice variants of the genes including splice donor, acceptor, splice region (119), non-coding transcript exons (103), 8 stop-gained, stop-lost SNP (1), NMD-transcript variants (20), and 616 were missense variants (Fig. 2).

### Selection of lethal nsSNPs

**Fig. 3:**
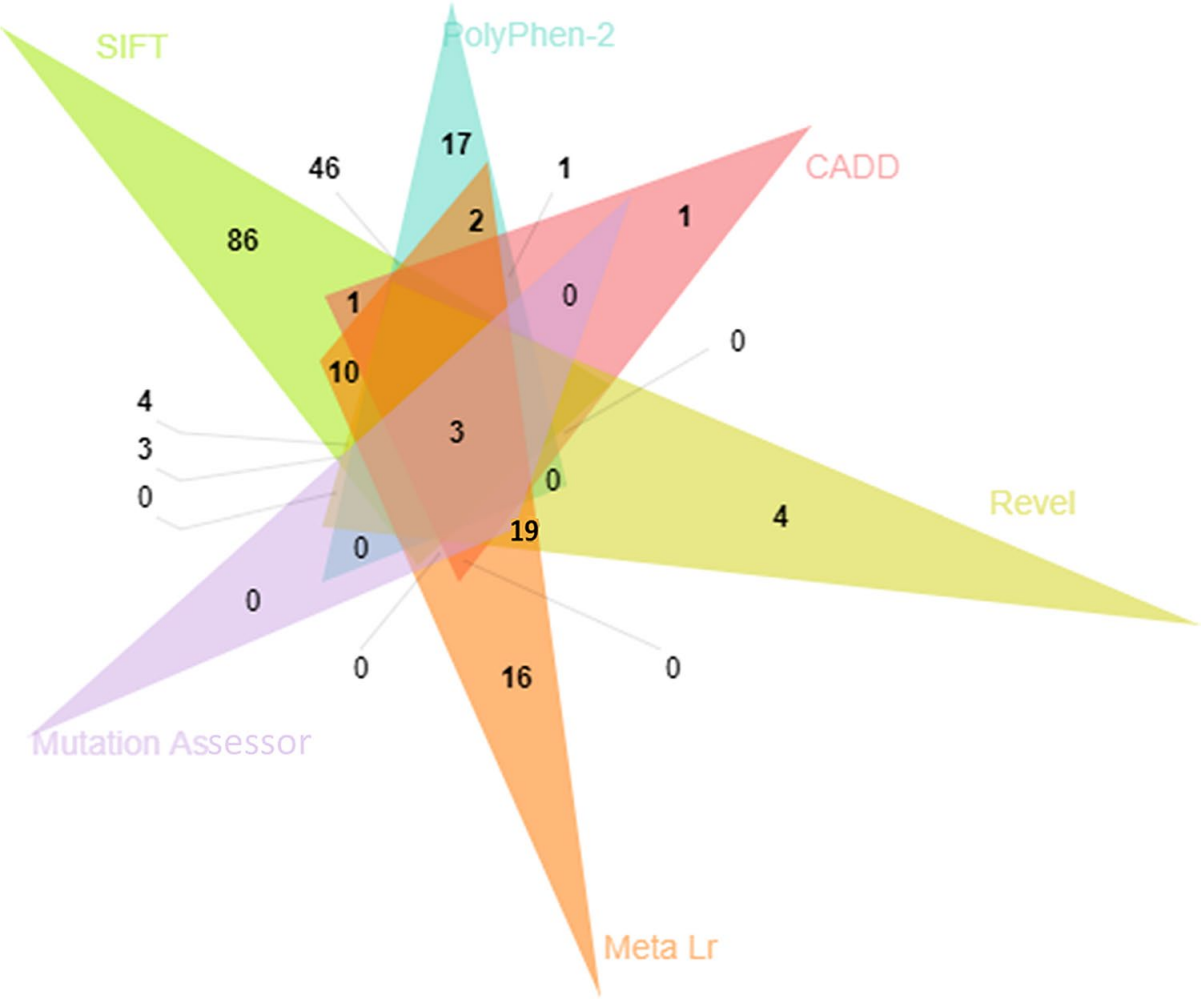
Pathogenicity predictions showing common deleterious non-synonymous SNPs

Among 616 missense SNPs, SIFT analysis predicted 248 SNPs (40.25%) as “deleterious”, however, the prediction rate of mutation by PolyPhen-2 was 149 (24.18%) as “probably damaging”. CADD, Revel, Meta LR, and Mutation Assessor reported 27 SNPs (4.38%), 109 SNPs (17.69%), 116 SNPs (18.83%), and 464 SNPs (6.49%) as likely deleterious, likely disease-causing, damaging, and high, respectively (Fig. S3). A total of six diverse bioinformatic resources, such as CADD, Mutation assessor, SIFT, Revel MetaLR, and PolyPhen-2 collectively showed three lethal missense variants (Fig. 3); *A2M* rs201769751, rs778604418, and *PARP1* rs193238922 (Table S1).

### Protein modeling and mutation effect on protein stability

**Fig. 4:**
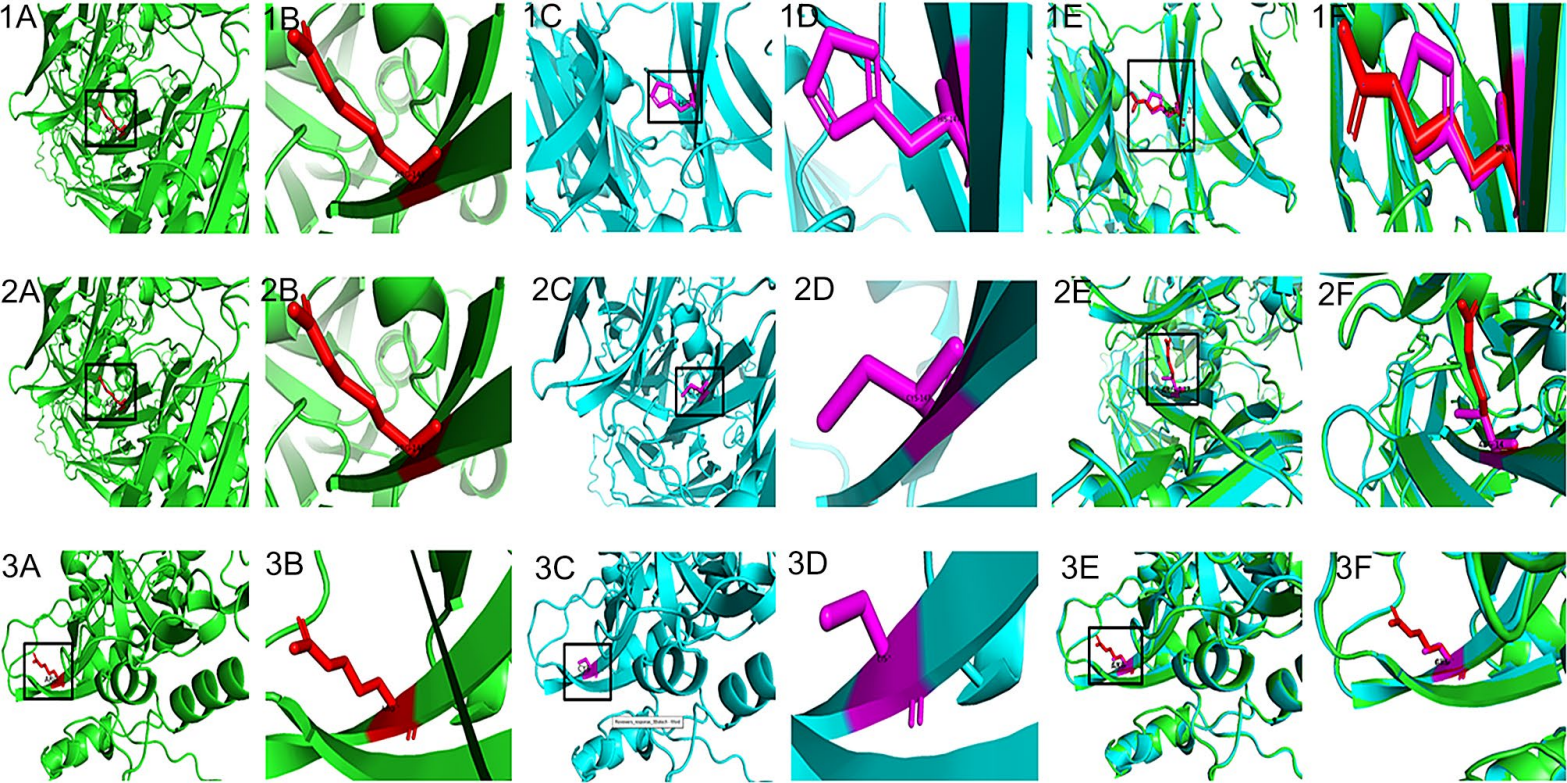
Native, mutant and superimposition of native and mutant modeled structures of the A2M (1) rs201769751 (2) rs778604418 (3) PARP1 rs193238922. **a** Structure of native protein, **b** enlarged structure of native protein, **c** structure of mutant protein, **d** enlarged structure of mutant protein, **e** superimposed model of native and mutant protein structures, **f** enlarged superimposed model of native and mutant protein structures

Out of three deleterious SNPs identified, A2M (rs778604418) and PARP1 (rs193238922) showed a change in hydrophobicity or hydrophilicity, but none of them showed a change in its polarity. The change in polarity and hydrophobicity may affect the protein structure and its activity. The divergence in free energy of unfolding between native form and mutant form of proteins known as ΔΔ*G* is calculated by CUPSAT tool using structural environment-specific atom capability and torsion angle capability. Henceforth, the stability of the protein was identified in terms of predicted ΔΔ*G* values (kcal/mol). Out of three deleterious SNPs, A2M (rs778604418) showed more stability with a predicted ΔΔ*G* value of 3.35 kcal/mol and A2M (rs201769751), PARP1 (rs193238922) affects the protein stability with predicted ΔΔG value of − 5.07 kcal/mol and − 0.51 kcal/mol, respectively (Table S2). The native form of the protein A2M (AlphaFold ID: AF-P01023-F1), PARP1 (AlphaFold ID: AF-P09874-F1) was retrieved from the AlphaFold database, and the mutant form was modeled and validated using the Ramachandran plot. The mutant model showed that 95% of the amino acids were present in the favorable region and considered for further in silico analysis. The native and mutant protein forms of deleterious SNPs along with overlapping models were shown (Fig. 4). A high QMEAN score and sequence identity from the swiss model was considered for the superimposition of the mutant model over the native structure and visualized using Swiss-PDB Viewer. The total energy of mutant structures in all three polymorphisms was less compared to native protein structures. Hence, it is believed that these three deleterious SNPs may affect the protein structure and function. Further, the calculated RMSD value for A2M (rs201769751, rs778604418) and PARP1 (rs193238922) were 0.052 Å, 0.047 Å, and 0.221 Å, respectively. It is reported that the higher the RMSD value, the greater the deviation between the native and mutant forms of the protein structures, which in turn indicates the change in its functional activity. The total energy and RMSD value of native and mutant forms of all the polymorphisms are tabulated in Table S3.

### Prediction of functional microRNA target SNPs

The functional microRNA targeting SNPs were predicted using three different resources, and these were miRNASNP3, PolymiRTS, and MirSNP, which concomitantly reported 12 SNPs (rs11552192 in the *BECN1*, rs60393216 in the *GSK3B,* rs9903 in the *MAP1LC3B,* rs1065154, and rs10277 in the *SQSTM1,* rs10415095 in the *STK11,* rs28382747, rs28382755, rs28382752, rs28382740, rs28382742, rs17330644 in the *XIAP*) with the minor allele frequency (MAF) of 10% in the microRNA binding sites. It also indicates any miRNAs linked with SNPs residing in the target position would create or destroy a miRNA-mRNA binding site (Table 2).

**Table 2:** Details of miRNA target site SNPs with minor allele frequency > 0.1

Sl no.	Gene	Common SNPs among MirSNP, miRNASNP3 and PolymiRTS databases	MAF	miRNA binding at mutant allele	miRNA binding at ancestral allele	Effect
1	*BECN1*	rs11552192	0.11	hsa-miR-590-3p	–	Create
2	*GSK3B*	rs60393216	0.30	hsa-miR-3662	–	Break
3	*MAP1LC3B*	rs9903	0.18	–	hsa-miR-3960	Break
				–	hsa-miR-4467	Break
				–	hsa-miR-4484	Break
4	*SQSTM1*	rs1065154	0.277	–	hsa-miR-4643	Create
				–	hsa-miR-466	Create
				hsa-miR-4694-3p	–	Break
				–	hsa-miR-4717-3p	Create
				–	hsa-miR-4802-3p	Create
		rs10277	0.31	hsa-miR-3178	–	Break
				hsa-miR-4634	–	Break
5	*STK11*	rs10415095	0.29	hsa-miR-4781-5p	–	Break
				–	hsa-miR-330-3p	Create
6	*XIAP*	rs28382747	0.26	hsa-miR-4634	–	Break
		rs28382755	0.26	hsa-miR-143-5p	–	Create
				hsa-miR-5693	–	Create
		rs28382752	0.26	–	hsa-miR-150-5p	Create
				–	hsa-miR-562	Create
				–	hsa-miR-5697	Create
		rs28382740	0.26	–	hsa-miR-24-1-5p	Break
				–	hsa-miR-24-2-5p	Break
				–	hsa-miR-625-3p	Break
		rs28382742	0.26	–	hsa-miR-326	Break
				–	hsa-miR-330-5p	Break
				hsa-miR-3675-5p	–	Create
				hsa-miR-5195-5p	–	Create
		rs17330644	0.26	hsa-miR-3609	–	Create
				hsa-miR-548ah-5p	–	Create
				–	hsa-miR-5586-3p	Break

### SNPs at the transcription factor binding site (TFBS)

A sum of 22 SNPs was found to be in TFBS with MAF > 0.1 by SNP2TFBS; among them, 17 and 5 SNPs reside in the upstream and 5′-UTR region, respectively. Further, SNPInspector projected that rs712829 in the *EGFR* generates 15 TFBS; rs60221525 in the *BECN1*, rs4645980 in the *CASP9*, and rs5393 in the *SLC2A2* impaired binding position for 14 transcription factors (TFs). The effect of 22 SNPs at TFBS revealed those SNPs that would generate or disrupt the positions for the binding of TFs (Table 3).

**Table 3:** Impact of SNPs in the transcription factor binding site with MAF > 0.1

Sl no.	Gene name	rsID	Allele	Function	MAF	Generated sites	Deleted sites
1	*A2M*	rs226380	A > C	UTR5	0.47	SMAD	SORY, GREF, GREF
2	*APP*	rs364048	T > C	Upstream	0.13	LEFF, HNFP	BCDF, FKHD
3	*ATG7*	rs2594971	G > A/C	Upstream	0.49	NOLF, MZF1, E2FF, XCPE, MZF1, AP2F, GCF2, ZTRE	CTCF, NOLF, ZF02, ZTRE, GLIF, EGRF, SP1F, AP2F
4	*BECN1*	rs60221525	C > A/G	Upstream	0.10	ZF07, ZF02, BEDF, GLIF, INSM, CNBP, RREB, GCMF	MAZF, ZF07, NDPK, ZF02, BEDF, SP1F, CTBP, KLFS, EGRF, GLIF, INSM, CNBP, RREB, NDPK
5	*CASP3*	rs114746204	G > C/T	Upstream	0.39	MAZF, KLFS, ZTRE, NDPK, EGRF, GLIF, ZF02, CTCF, SP1F, ZF07, E2FF, PURA, HEAT	MOKF, SP1F, BTBF, BTBF
6	*CASP9*	rs4645978	C > A,T	Upstream	0.41	CEBP, GCMF, ZF30, MAZF, NR2F, PERO, ZF07	MAZF, PLAG, PAX6, SP1F, KLFS, ZTRE, E2FF, NDPK, EGRF, IKRS, SPZ1, ZF37
7	*CASP9*	rs4645980	C > A,T	Upstream	0.41	CTBP	MAZF, PLAG, SP1F, KLFS, ZTRE, E2FF, NDPK, ZF42, ZF02, ZF5F, BEDF, GCF2, XCPE, EGRF
8	*DDIT3*	rs703835	A > G	UTR5	0.21	HUB1, STAT, CEBP, RBPF, IKRS	STAT, CEBP
9	*EGFR*	rs712829	G > C/T	UTR5	0.22	MAZF, EGRF, KLFS, CTCF, VEZF, ZF02, SP1F, NDPK, CTBP, MZF1, ZF07, BEDF, GLIF, CTBP, INSM	SP1F, KLFS, ZF02, BEDF, GLIF
10	*EIF4EBP1*	rs3750243	C > A/G	Upstream	0.23	GCF2, CTCF, ZTRE, KLFS, MAZF, E2FF, NDPK, EGRF	KLFS, BEDF, ZF57, EBOX, HESF, CHRE
11	*IL1B*	rs1143627	G > A	Upstream	0.47	PTBP, MYT1	–
12	*INS*	rs3842737	T > G	Upstream	0.11	PLAG, RXRF, ZF02, SP1F, ZF07, BEDF, KLFS, ZF37	ZF02, TAIP
13	*INS*	rs689	A > G,T	UTR5	0.35	PAX6, GREF, PBXC, RXRF, TALE, MYRF	TF3A, NGRE, IKRS
14	*MAPK3*	rs61764202	C > T	Upstream	0.36	AP1F	SP1F, KLFS, ZTRE, E2FF, HESF
15	*PARP1*	rs2793379	T > A/C	Upstream	0.20	PARF, TALE, HAND, EBOX, MYOD, NREB	PDX1
16	*PARP1*	rs2077197	C > G/T	Upstream	0.26	CDXF, ABDB, E2FF, EGRF, ZF02, HBOX, RXRF	PLAG, ZF02, GLIF, RREB
17	*RPS6*	rs35096177	A > G	Upstream	0.12	SORY, CLOX, DMRT, HNF6, HOXC, LEFF, AP1F	SORY, ARID, HOXF, PIT1, BRNF, CART, LHXF, HOXF, LHXF, HBOX, HOMF, NKX1, NKX6
18	*SLC2A2*	rs5393	T > G	Upstream	0.22	NOLF, NGRE, BCDF	LHXF, DLXF, PDX1, BRN5, BRNF, HOMF, GATA, NKX1, OCT1, CART, HOXF, NKX6, PAXH, DLXF
19	*SLC2A2*	rs5394	G > A	Upstream	0.14	MIPU	TF3A, STAF
20	*STK11*	rs3795061	G > C	Upstream	0.15	CTCF, ETSF	ZF02, GCMF, KLFS
21	*TP53*	rs2909430	C > G/T	UTR5	0.15	ZF01, IRXF, RP58, CEBP	PAX6
22	*XIAP*	rs12687176	C > T	Upstream	0.22	MAZF, E2FF, SPZ1	MAZF, GLIF, ZF07, SAL4

*MAF* minor allele frequency

### SNPs in enhancers

The two databases, namely, EnhancerDB and HaploReg were employed to identify SNPs in the enhancers which unanimously identified 42 SNPs residing in the introns and 1 3′-UTR SNP with MAF > 0.1. Out of 43 SNPs, rs3795063 in the *STK11* gene showed 19 regulatory motifs that were altered which included CAC-binding-protein, CACD, E2A, Egr-1, Irf, Klf4, Klf7, Myc, Myf, NRSF, Pou2f2, Rad21, SMC3, SP1, SP4, TATA, UF1H3BETA, YY1, and Zfp740. The rs10861203 in the *HSP90B1* gene reported 14 regulatory motifs that were altered and these included BCL, BDP1, ELF1, Egr-1, Ets, FEV, Myc, NERF1a, Nrf-2, Pax-5, STAT, TBX5, Tel2, and p300. The specifics of SNPs residing in the enhancers and their altered regulatory motifs are catalogued (Table 4).

**Table 4:** SNPs in enhancers and their altered regulatory motifs with MAF > 0.1

Sl no	Gene	Chromosome	rs ID	MAF	Reference Allele	Alternative Allele	Enhancer ID	Functional annotation	Regulatory motifs altered
1	*SLC2A1*	chr1	rs11210771	0.36	T	C	enh11804	Intronic	Rad21
2	*SLC2A1*	chr1	rs7512565	0.14	C	T	enh107379	Intronic	Irf, Maf, Mxi 1, Nkx2
3	*SLC2A1*	chr1	rs710221	0.43	G	A	enh107379	Intronic	HNF4
4	*ADIPOQ*	chr3	rs875571	0.34	T	C,G	enh48780	Intronic	BCL, CCNT2, GATA, HDAC2, HMGN3, TAL1
5	*ADIPOQ*	chr3	rs55647362	0.30	A	G	enh7748	Intronic	EBF, GR, Sox, TATA
6	*ATG7*	chr3	rs11915050	0.40	A	G	enh6998	Intronic	p300
7	*CASP9*	chr1	rs4646029	0.41	G	A	enh78963	Intronic	ERalpha-a, Pax-5, TCF11::MafG, ZID
8	*CASP9*	chr1	rs4233536	0.41	C	T	enh98427	Intronic	HEY1,Pou1f1,Sox
9	*DDIT3*	chr12	rs4759277	0.37	C	A	enh98973	Intronic	Znf143,p300
10	*EGFR*	chr7	rs6593207	0.18	T	C	enh24214	Intronic	Zfp691
11	*EIF4EBP1*	chr8	rs9644811	0.33	G	A	enh24927	Intronic	Dbx1, Evi-1, Foxa, Foxp1, HDAC2, HMG-IY, Ncx, TATA, Zfp105
12	*EIF4EBP1*	chr8	rs10958541	0.23	G	C	enh40918	Intronic	AhR::Arnt_1, Arnt, NF-E2, NRSF, Nanog, TATA
13	*ERBB2*	chr17	rs2952155	0.37	T	C	enh17134	Intronic	BCL, Egr-1, Ets, GATA, Hsf, Maf, PU.1, Pax-5, STAT, UF1H3BETA, Zfp281, Znf143
14	*FXYD2*	chr11	rs869789	0.11	G	A	enh64534	3'-UTR	CTCF,TCF12
15	*GSK3B*	chr3	rs28536662	0.40	G	A	enh20841	Intronic	EWSR1-FLI1, Gfi1, HDAC2, HMG-IY, Mef2
16	*GSK3B*	chr3	rs4688054	0.30	T	C	enh7475	Intronic	CTCF, ERalpha-a, Foxk1, Irf, Rad21, SMC3, SZF1-1
17	*HK2*	chr2	rs1545522	0.30	T	C	enh5656	Intronic	MIF-1
18	*HK2*	chr2	rs3771763	0.12	C	T	enh18686	Intronic	ERalpha-a, GCNF, HNF4, RXRA, SF1
19	*HSP90AA1*	chr14	rs7156564	0.32	A	G	enh15961	Intronic	Mef2
20	*HSP90AA1*	chr14	rs1746587	0.10	A	G	enh3457	Intronic	Dobox4
21	*HSP90B1*	chr12	rs312136	0.24	A	G	enh29941	Intronic	AIRE
22	*HSP90B1*	chr12	rs7980326	0.44	T	G	enh14948	Intronic	Myc,Smad3,Zfp410
23	*HSP90B1*	chr12	rs10861203	0.19	G	A	enh86141	Intronic	BCL, BDP1, ELF1, Egr-1, Ets, FEV, Myc, NERF1a, Nrf-2, Pax-5, STAT, TBX5, Tel2, p300
24	*HTRA2*	chr2	rs13411185	0.29	C	T	enh33451	Intronic	Pax-5
25	*HTRA2*	chr2	rs72920676	0.19	C	T	enh81722	Intronic	AP-1, Mef2, NRSF, YY1
26	*HTRA2*	chr2	rs17838412	0.11	T	C	enh33456	Intronic	Foxp3,Pou5f1
27	*IGF1R*	chr15	rs6598541	0.44	A	G	enh16457	Intronic	NRSF,RFX5
28	*ITGB1*	chr10	rs10763923	0.40	G	A	enh43272	Intronic	Cdx2,Hoxd10
29	*ITGB1*	chr10	rs11009338	0.17	A	G,T	enh62030	Intronic	EWSR1-FLI1,TAL1,VDR
30	*MAP1LC3B*	chr16	rs4598916	0.43	C	G	enh32021	Intronic	DBP,Irf,PU.1
31	*MAP1LC3B*	chr16	rs8052244	0.16	G	C	enh32020	Intronic	BCL, ELF1, Egr-1, Ets, GATA, Maf, NERF1a, PU.1, Pax-5, SPIB, TEF, TFIIA, p300
32	*MAP1LC3B*	chr16	rs3794673	0.24	G	C	enh115621	Intronic	Ets,PLAG1,Pax-6
33	*NOS2*	chr17	rs4796222	0.21	A	G	enh17068	Intronic	Sox
34	*RPS6*	chr9	rs944720	0.29	A	G	enh25490	Intronic	Foxf2, Foxl1, Foxp3, Gm397, Nanog, Pou1f1, Pou2f2, Pou3f3
35	*RPS6KB1*	chr17	rs11079374	0.20	T	C	enh17258	Intronic	LBP-1,LBP-9
36	*RPS6KB1*	chr17	rs9910598	0.10	G	A	enh96758	Intronic	Msx-1,Ncx
37	*SERPINB5*	chr18	rs11661184	0.25	G	A	enh4892	Intronic	CTCF, HNF4, RXRA, Rad21, SMC3, SP1, TATA
38	*SOD2*	chr6	rs6913904	0.11	A	G	enh9509	Intronic	Ets, Pax-4, Pbx3, TBX5
39	*SQSTM1*	chr5	rs502729	0.49	A	C	enh22874	Intronic	Osr,TCF12
40	*SQSTM1*	chr5	rs59203082	0.17	C	T	enh22871	Intronic	Foxp1,RFX5
41	*SQSTM1*	chr5	rs10464093	0.42	G	A	enh22874	Intronic	Sin3Ak-20,TATA
42	*STK11*	chr19	rs3795063	0.25	C	G,T	enh17841	Intronic	CAC-binding-protein, CACD, E2A, Egr-1, Irf, Klf4, Klf7, Myc, Myf, NRSF, Pou2f2, Rad21, SMC3, SP1, Sp4, TATA, UF1H3BETA, YY1, Zfp740
43	*STK11*	chr19	rs34928889	0.46	G	A	enh17841	Intronic	ERalpha-a,Rad21,Zfx

*MAF* minor allele frequency, *chr* chromosome

## Discussion

Detection of therapeutically effective entity counter to the disease from a pre-existent molecule repository may substantially reduce the time and efforts against new drug discovery and clinical trial randomization. The approach of repurposing the existing drugs has resulted in the detection of a large number of effective molecules for the treatment of COVID-19 infection (Ciliberto et al. 2020).

In order to simplify the overview of large number of 2-DG interacting genes that has been extracted, massive number of SNPs residing in respective genes were mined and characterized based on their location. The distribution of these SNPs was depicted by circos which is an unambiguous representation to lessen the inherent complexities and consider the density and dynamic range within huge data sets (Krzywinski et al. 2009). Further, our in silico approach has detected 80 genetic variants associated with 2-DG interacting genes using diverse bioinformatics resources. Therefore, an assessment of these variants was performed by employing various SNP prediction resources and by choosing the overlapping SNPs to overcome the false-positive findings. The pathway analysis aids in investigating interrelationships of terms and functional groups that constitute biological networks (Bindea et al. 2009). The pathway analysis of 2-DG interacting genes emphasized various processes: cytochrome C-mediated apoptotic response, interleukin-4, and interleukin-13 signaling, among others. Interestingly, the assessment also indicated susceptible gene loci for SARS-CoV infections. The pathway assessment among 2-DG interacting genes also highlighted apoptosis-related signaling mediated by the caspase family of proteins which may modify the metabolism of cells and enhance the rate of cell death (Gioti et al. 2021) and its potential role in viral infection inhibition (Plassmeyer et al. 2021). Cell death due to 2-DG in various tumor cells has been reported and could be mediated by ER stress/autophagy in HCT116 colon cancer cells or through Cytochrome C-Caspase 3-PARP axis in certain other cells (Maximchik et al. 2018). Similarly, A2M which is a key anti-inflammatory protease can induce cell proliferation when ligated to chaperon GRP78 by increasing the glucose uptake (Vandooren and Itoh 2021). GRP78 also accumulates upon ER stress induced by 2-DG thus sequentially increasing its uptake when provided in place of glucose (Kim et al. 2018). Thus, any structural alterations in A2M may determine the efficacy of 2-DG treatment.

Often 3′-UTR and less frequently exon bound miRNAs silence and regulate the genes at a posttranscriptional level. The variations due to SNPs introduced into the miRNA binding regions may diminish binding affinity and consequently affect its function (Prabhu et al. 2021). We extracted the SNP information of 2-DG interacting genes to unravel the miRNA binding sites employing three databases namely miRNASNP3, MirSNP, and PolymiRTS and examined whether or not miRNAs linked polymorphisms residing in the target region would generate or disrupt a miRNA-mRNA binding region. The findings of our study showed the impact of two miRNA target SNPs (rs1065154, rs10277) residing in the *SQSTM1* gene which could create or break at ancestral and mutant allele. Expression quantitative trait loci analysis is a robust technique toward determining genetic loci linked with quantitative variations in gene expression. After employing Genome-Wide Association analysis to the set of records containing approximately 3,00,000 SNPs and 48,000 mRNA expression traits from high throughput technique, researchers found 1226 significant associations, out of which 95 associations were linked to ADME of drugs. The variant rs10277 residing in the gene *SQSTM1* in human liver samples reported that allele C is linked with increased transcription compared to allele T. These data broaden our understanding regarding the genetic features of inter-individual variation in gene expression in conjunction with specific prominence on pharmacogenomics (Table S4) (Schröder et al. 2011; Whirl-Carrillo et al. 2012).

In this study, the influence of polymorphisms in *TFBS* and enhancers were also analyzed. The massive number of genetic variants detected from GWAS resides in the genome’s noncoding region and are of significant interest when located in regulatory sites such as promoters and enhancers as these variants may influence gene expression and these may play a major role in the complex traits that elicits drug response. Thus, we screened the 5′-UTR and upstream SNPs of the selected genes to verify whether the substitution of SNP allele and modified TF binding sites would possibly perturb gene regulation (Buroker 2017).

Pathogenic and other exposures cause leucocytes to respond quickly, with effects ranging from cytokine generation to migration and engulfing by phagocytosis (Marsin et al. 2002; Yang et al. 2012; Wahl et al. 2012). Activation of mononuclear cells with lipopolysaccharide enhanced the production of cytokines IL-1B, IL-6, and TNF-alpha, as predicted (Fangradt et al. 2012; Freemerman et al. 2014). Accelerated glycolytic flow produces ATP quickly to meet these critical processes, which are bioenergetically expensive (Palsson-Mcdermott and O’Neill 2013; Macintyre and Rathmell 2013). For all three cytokines, the competitive glycolysis inhibitor 2-DG dramatically inhibited lipopolysaccharide-mediated generation of cytokines (Jones et al. 2015). Our findings reported rs1143627 residing in *IL1B* generated two TFBS for PTBP, MYT1. One of the studies proclaimed that rs1143627 residing in the gene *IL1B* was found to be associated with Influenza A susceptibility in humans. The findings also showed that aged adults or individuals of any age with comorbid or immunosuppressive conditions might be at a greater risk of disease development. *IL1B* rs1143627 was also considered to be susceptibility alleles in individuals suffering from liver fibrosis infected by the hepatitis B virus (Wu et al. 2018). Extensive data reported the role of two variants, namely, rs712829 residing in *EGFR* gene and rs1143627 in *IL1B* gene in NCI-60 cancer cell lines and human samples, highlighting the effect of genotype on neoplasms and psoriasis on the usage of diverse drugs molecules (Tables S4, S5) (Whirl-Carrillo et al. 2012). Additionally, enhancers that regulate gene expression function as rheostats for transcription, which will further tune up the levels of specific transcripts (Corradin and Scacheri 2014). Henceforth, in the current study 43 SNPs have shown a wide spectrum of altered motifs that may result in gene regulation.

Due to the complexity of the infection, an apt determinative model and efficacious medication for COVID-19 infection are yet to be evolved. As the innate immune system is inadequate to produce a powerful immune response counter to the virus, multi-targeted factors that mitigate viral infection, replication, and host immune reactions are warranted. In the present study, a sum of three polymorphisms (*SQSTM1* rs10277, *IL1B* rs1143627, *EGFR* rs712829) of 2-DG interacting genes may increase the susceptibility to SARS-CoV infections than other polymorphisms. However, these identified polymorphisms need to be considered by experimental validation of the likelihoods proposed in the current work is required in larger cohorts for repurposing the drug. Further, this in silico study was conducted to shed light on the pharmacogenomic concerns of 2-DG against SARS-CoV-2. We believe that the selected variants in the current study should be wisely considered to overcome adverse drug reaction and to strengthen the foundation for future medical exploration. Nevertheless, it is universally believed that an SNP acts through neighboring genes when it is most likely connected to a phenotype or illness. Therefore, it is undeniable that the present strategy may overlook certain associated genes.

## Conclusions

In the current in silico study, efforts were made to identify the genetic biomarkers of 2-DG interacting genes, which may determine the risk of gene polymorphisms and drug response. The in silico data mining strategy aids predominantly in finding the drug interacting genes, and their respective pathways and supports in assessing the influence of SNPs in distinct genic regions. Eventually, the information creates an integrated foundation to delineate the intricate molecular relationships among 2-DG interacting genes and may subsequently provide insight to predict COVID-19 infection risk and treatment strategies with 2-DG.

## Supplementary Information

Below is the link to the electronic supplementary material.

Supplementary file1 (PDF 580 KB)
