# Supplementary material for: Pharmacogenomics deliberations of 2-deoxy-d-glucose in the treatment of COVID-19 disease: an in silico approach
Source: 3 Biotech. 2022 Sep 21;12(11):287. doi: 10.1007/s13205-022-03363-4 (PMC9491670; doi:10.1007/s13205-022-03363-4)
Supplement: Supplementary file 1 — Supplementary file1 (PDF 580 KB) [file 13205_2022_3363_MOESM1_ESM.pdf]

Supplementary information

Pharmacogenomics Deliberations of 2-Deoxy-D-Glucose in the Treatment of COVID-19 Disease: An *In silico* Approach

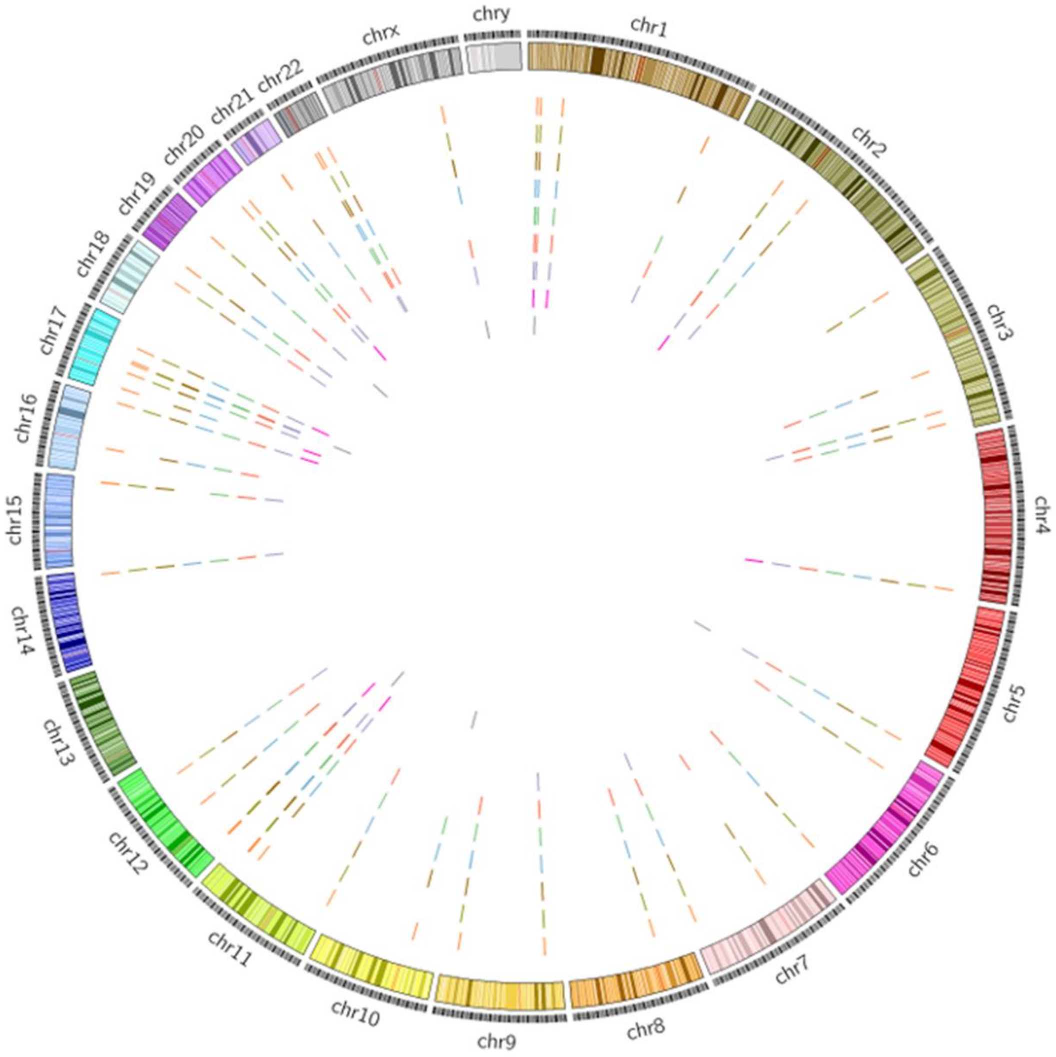

Fig. S1 Circos plot representing SNP distribution across 48 interacting genes of 2-DG

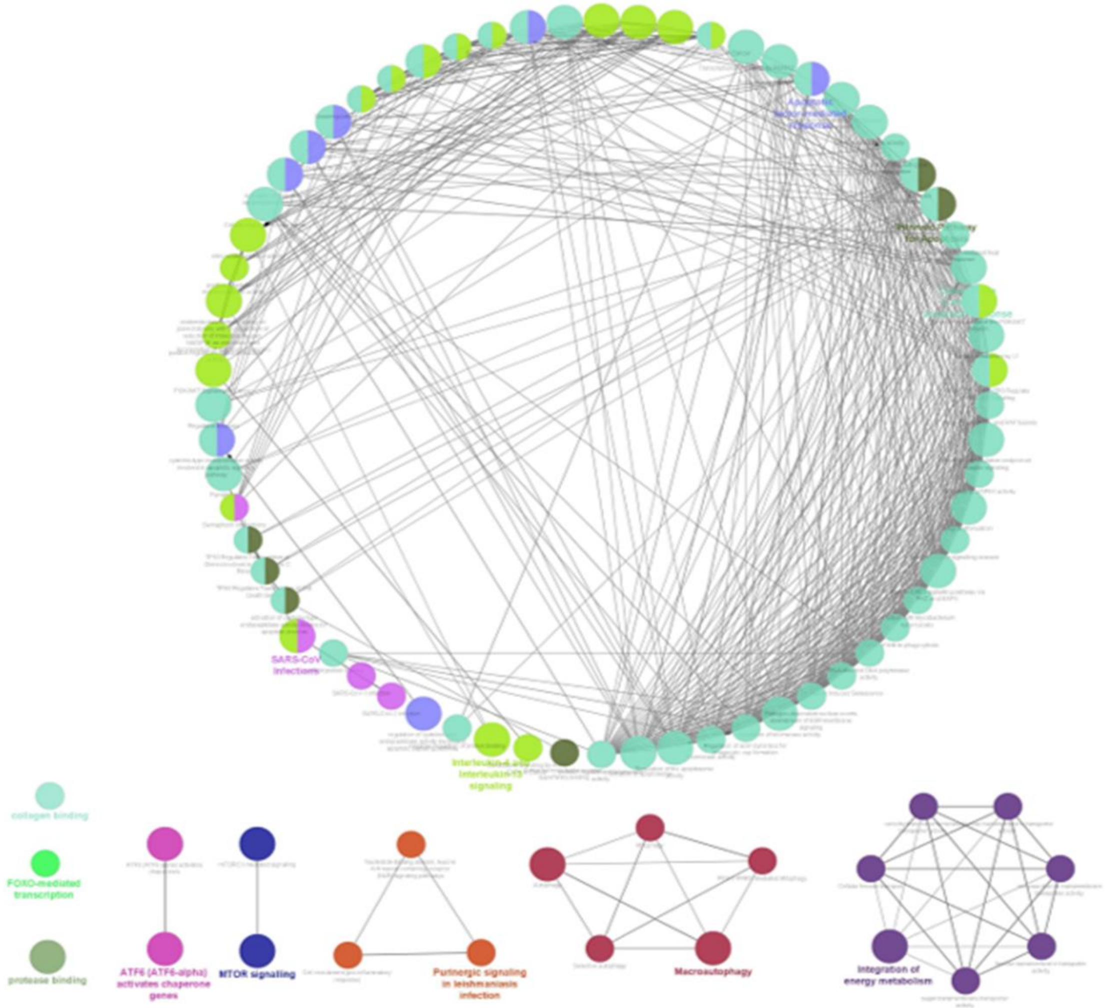

Fig. S2 Pathway interaction of 2-DG interacting genes

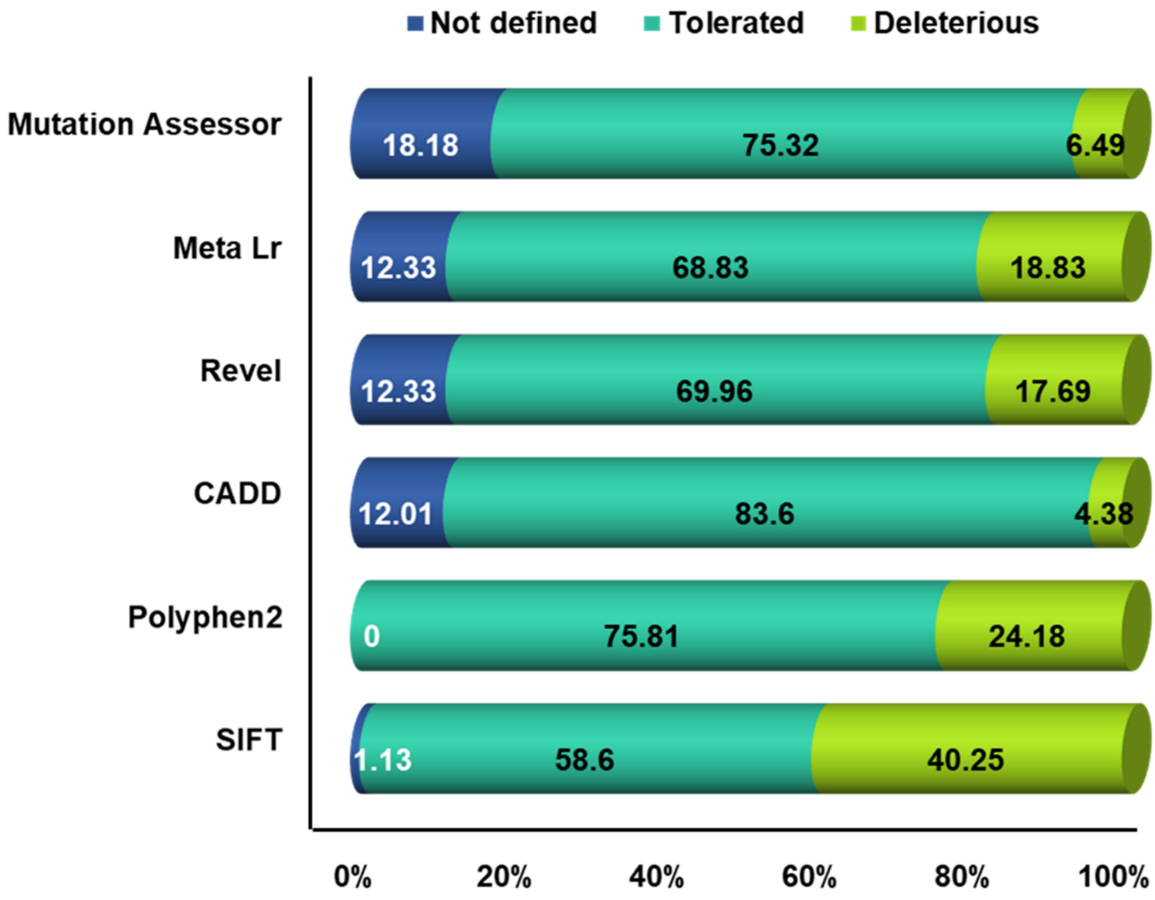

Fig. S3 Characterization of functional SNPs in 2-DG interacting genes

Table S1. Deleterious nsSNPs and associated amino acid change

Sl no.	Gene	Gene symbol	MAF	rsID	Chromosome	Observed alleles	Minor allele	Amino acid change
1	*Alpha-2-Macroglobulin*	*A2M*	0.000599	rs201769751	Chr12	C/T	T	Arg to His
2	*Alpha-2-Macroglobulin*	*A2M*	0.0002	rs778604418	Chr12	G/A	A	Arg to Cys
3	*Poly(ADP-Ribose) Polymerase 1*	*PARP1*	0.0002	rs193238922	Chr1	G/A	A	Arg to Cys

MAF minor allele frequency, chr chromosome

Table S2. Polarity, hydrophobicity, and hydrophilicity of the reported deleterious nsSNPs and stability of the protein

Sl no.	Gene	SNP ID	MAF	Mutation	Polarity	Change in polarity	Hydropathy index	Change in hydrophobicity/ hydrophilicity	Overall stability	Predicted ΔΔG (kcal/mol)
1	*A2M*	rs201769751	0.000599	R147H	Polar to Polar	No	-4.5 to -3.2	No	Destabilizing	-5.07
2	*A2M*	rs778604418	0.0002	R147C	Polar to Polar	No	-4.5 to 2.5	Yes	Stabilizing	3.35
3	*PARP1*	rs193238922	0.0002	R138C	Polar to Polar	No	-4.5 to 2.5	Yes	Destabilizing	-0.51

SNP single nucleotide polymorphism, *MAF* minor allele frequency, *A2M*-Alpha-2-macroglobulin, *PARP1*-Poly [ADP-ribose] polymerase 1

Table S3. Total energy (wild and mutant type), change in energy and RMSD value of the reported deleterious nsSNPs

Sl no.	Gene	SNP ID	MAF	Total Energy (Wild Type protein)	Total Energy (Mutant protein)	Change in Energy	Root Mean Square Deviation (Å)
1	*A2M*	rs201769751	0.000599	-63104.770	-55135.262	-7969.508	0.052
2	*A2M*	rs778604418	0.0002	-63104.770	-56169.379	-6935.391	0.047
3	*PARP1*	rs193238922	0.0002	-36999.613	-32789.559	-4210.054	0.221

SNP single nucleotide polymorphism, *MAF* minor allele frequency, *A2M*-Alpha-2-macroglobulin, *PARP1*-Poly [ADP-ribose] polymerase 1

Table S4. Details of variant annotations of selected 2DG interacting gene variants

Variant	Molecules	Genes	Association	P-value	Effect	Inference
rs10277	-	*SQSTM1*	Allele C is associated with increased transcription of SQSTM1 in human liver samples as compared to allele T	-	Metabolism/PK	-
rs712829	EGFR inhibitors	*EGFR*	Allele T is associated with cytotoxicity of egfr inhibitors	-	Toxicity	When combining the rs712829 variant and rs2227983 (R497K) variants, a weak association with drug cytotoxicty was observed in NCI60 cell lines
rs712829	Topoisomerase I inhibitors	*EGFR*	Genotypes GT + TT are associated with decreased sensitivity when treated with topoisomerase I inhibitors as compared to genotype GG	-	-	This was done in NCI-60 cancer cell lines
rs712829	Gefitinib	*EGFR*	Genotypes GT + TT are associated with increased response rate (RR) when treated with gefitinib in people with carcinoma, non-small-cell lung as compared to genotype GG	0.01	Efficacy	-

rs712829	Alkylating Agents	*EGFR*	Genotypes GT + TT are associated with decreased sensitivity when exposed to alkylating agents as compared to genotype GG	-	-	Done in NCI-60 cancer cell lines
rs712829	Gefitinib	*EGFR*	Genotypes GT + TT are associated with increased progression-free survival (PFS) time when treated with gefitinib in people with carcinoma, non-small-cell lung as compared to genotype GG	0.005	Efficacy	-
rs712829	-	*EGFR*	Genotype TT is associated with increased expression of EGFR NCI60 cell lines	0.005	-	Data were extracted from the NCI60 database (MT1147)
rs712829	Erlotinib	*EGFR*	Genotypes GT + TT are associated with increased sensitivity when exposed to erlotinib as compared to genotype GG	-	Dosage	Done in NCI-60 cancer cell lines
rs712829	Cetuximab, Panitumumab	*EGFR*	Genotypes GT + TT is associated with increased survival when treated with cetuximab or panitumumab in people with colorectal neoplasms as compared to genotype GG	0.025	Efficacy	Patients with the GT and TT genotype had a higher median progression-free survival time (9.0 and 11.6 months) and overall survival time (19.6 and 27.3 months), as compared to

						those with the GG genotype (6.4 months and 10.9 months, respectively)
rs712829	Geldanamycin	*EGFR*	Genotypes GT + TT are associated with decreased sensitivity when exposed to geldanamycin as compared to genotype GG	-	-	Done in NCI-60 cell lines
rs712829	Cetuximab, Panitumumab	*EGFR*	Allele T is not associated with response to cetuximab or panitumumab in people with colorectal neoplasms as compared to allele G	0.331	Efficacy	Meta-analysis with 3 studies. The authors did not provide the exact number of patients but stated that "the median number of patients per analysis was 110 (range 50 - 740)". Most definitions of response were variations of the RECIST criteria

rs712829	Cetuximab, Panitumumab	*EGFR*	Genotype GG is not associated with response to cetuximab or panitumumab in people with colorectal neoplasms as compared to genotypes GT + TT	0.089	Efficacy	No significant difference in disease control rate was seen between the two genotype groups. Using the response evaluation criteria in solid tumors (RECIST), disease control rate was the percentage of patients with complete response, partial response or stable disease (as opposed to patients with progressive disease)
rs712829	Erlotinib	*EGFR*	Genotype GG is associated with increased severity of diarrhea when treated with erlotinib in people with neoplasms as compared to genotypes GT + TT	0.009	Toxicity	Similar associations were found when comparing with the -216/-191 diplotypes or haplotypes
rs1143627	Tumor necrosis factor alpha (TNF-alpha) inhibitors, ustekinumab	*IL1B*	Genotypes AG + GG is associated with decreased response to tumor necrosis factor alpha (TNF-alpha) inhibitors or ustekinumab in	0.0016	Efficacy	Good responders (those with a reduction of >=75% in PASI score after 3 months) were

			people with psoriasis as compared to genotype AA			compared against non-responders (those with a reduction of <50% in PASI score after 3 months). Statistical results adjusted for age, gender, psoriatic arthritis and previous treatments and corrected for multiple testing using the false discovery rate. Please note that alleles have been complemented to the plus chromosomal strand
rs1143627	Aspirin	*IL1B*	Allele A is associated with peptic ulcer when treated with aspirin in people with cardiovascular diseases as compared to allele G	-	Toxicity	The study did not discuss the direction of the association, but it might be a protective effect. Patients taking 100 mg aspirin. No p-value is reported

Table S5. Details of clinical annotations of selected 2DG interacting gene variants

Level	Variant	Gene	Molecules	Type	Phenotype
Level 3	rs712829	*EGFR*	Alkylating agents	Efficacy	Neoplasms
Level 3	rs712829	*EGFR*	Erlotinib	Toxicity	Neoplasms
Level 3	rs712829	*EGFR*	Erlotinib	Efficacy	Neoplasms
Level 3	rs712829	*EGFR*	Geldanamycin	Efficacy	Neoplasms
Level 3	rs712829	*EGFR*	Cetuximab, Irinotecan, Panitumumab	Efficacy	Colorectal neoplasms
Level 3	rs712829	*EGFR*	Gefitinib	Efficacy	Neoplasms
Level 3	rs712829	*EGFR*	Topoisomerase I inhibitors	Efficacy	Neoplasms
Level 3	rs1143627	*IL1B*	Aspirin	Toxicity	-
Level 3	rs1143627	*IL1B*	Tumor necrosis factor alpha (TNF-alpha) inhibitors, Ustekinumab	Efficacy	Psoriasis
